# Supplementary figures and images for: The Potential of Universal Primers for Barcoding of Subtropical Crops: Actinidia, Feijoa, Citrus, and Tea
Source: Int J Mol Sci. 2025 Jul 18;26(14):6921. doi: 10.3390/ijms26146921 (PMC12295962; doi:10.3390/ijms26146921)

# trnE/trnT

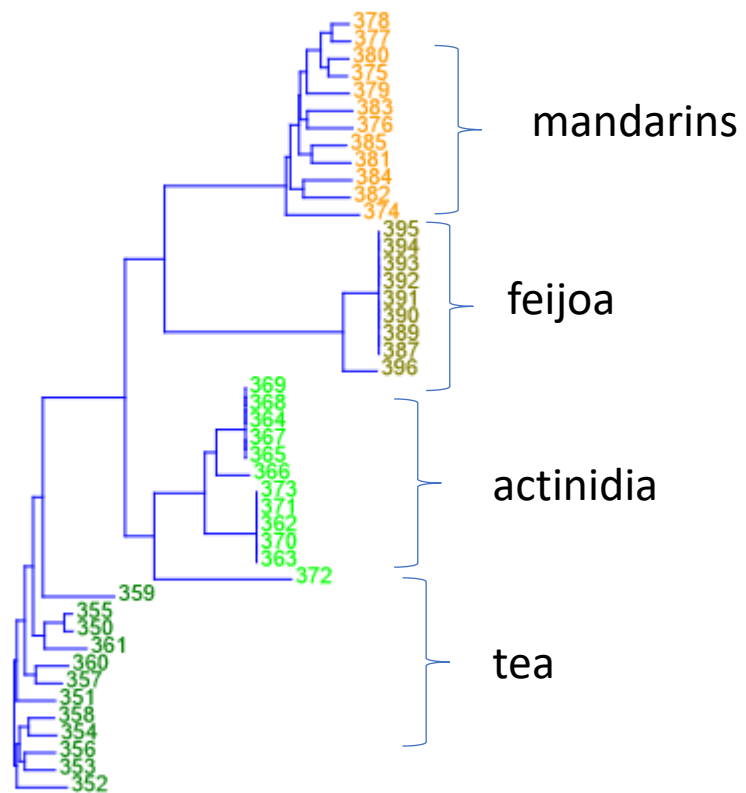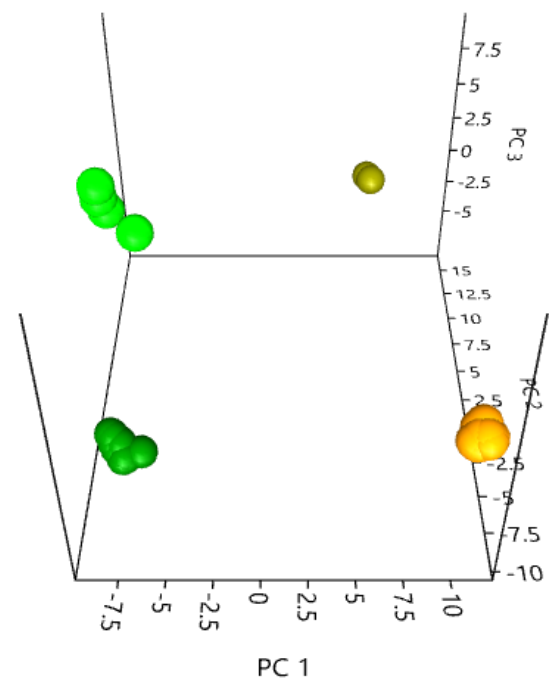

# Rpl23/rpi2.1

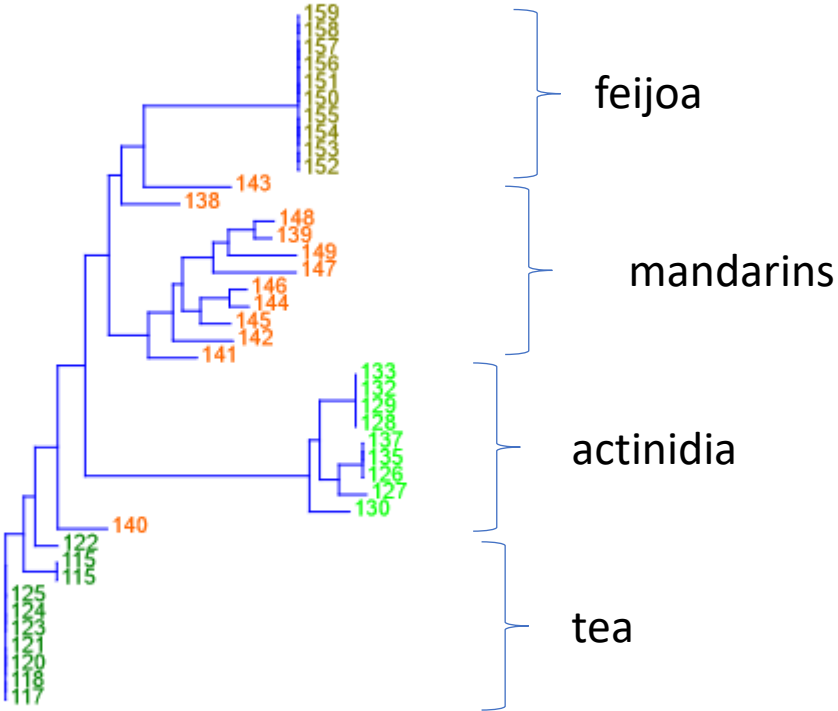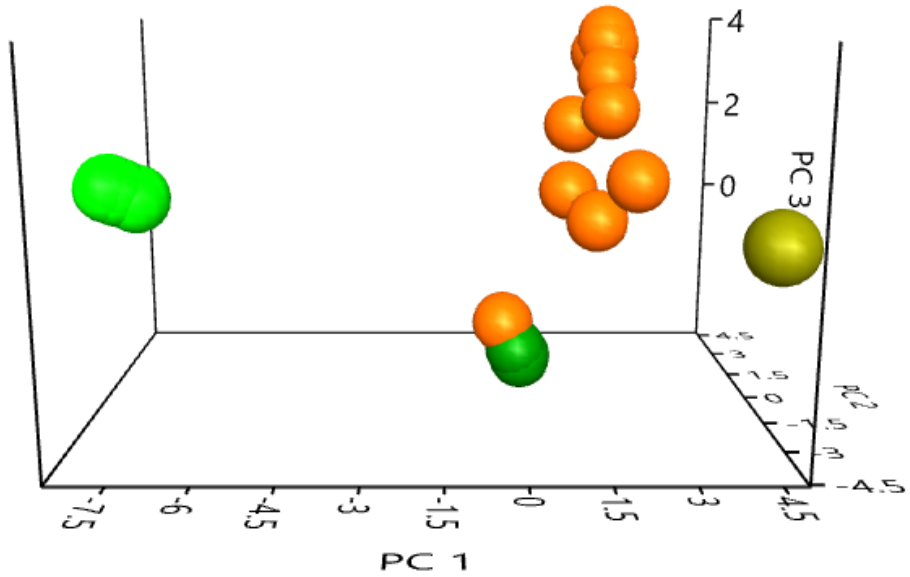

16S

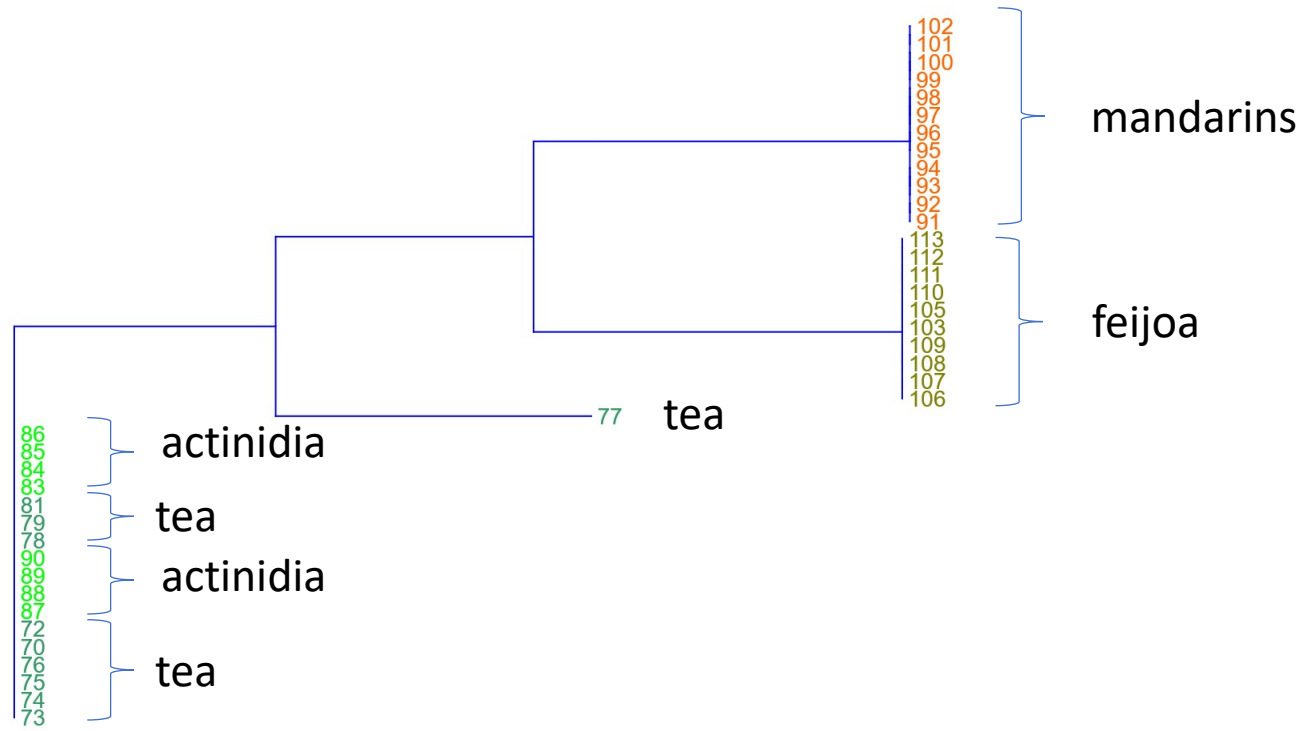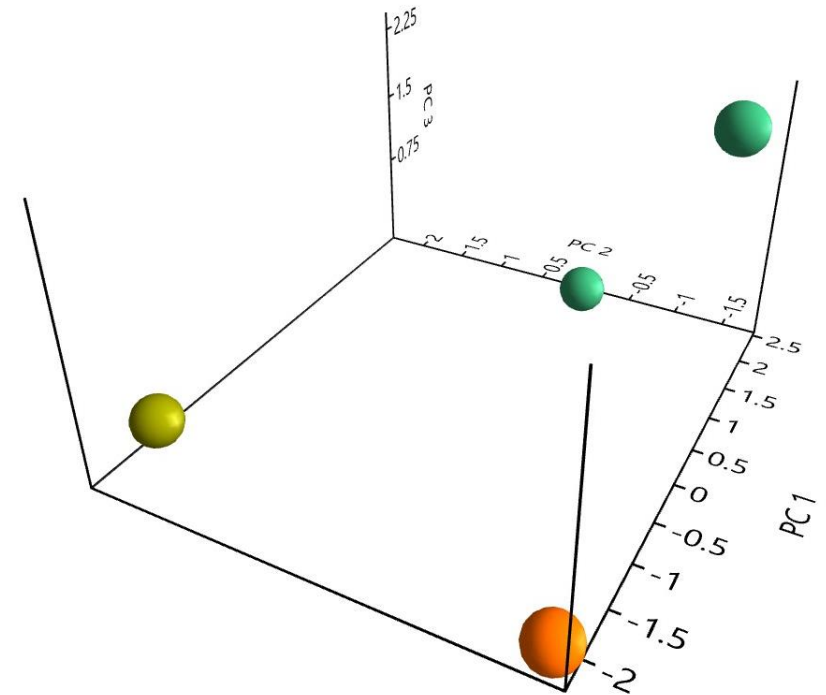

ITS-p3/ITS-u4

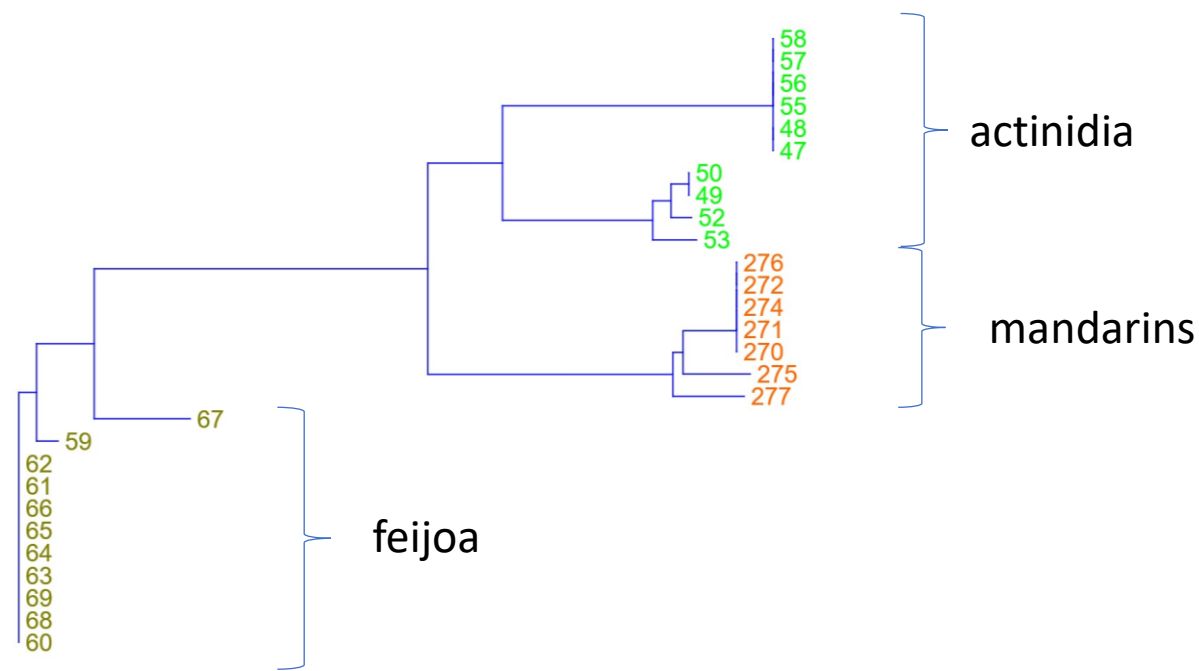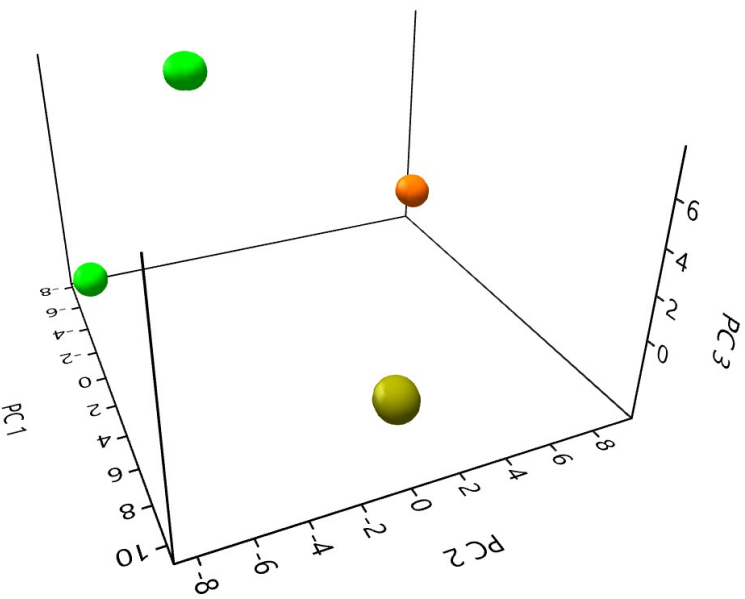

23S,4.5S/ 5S

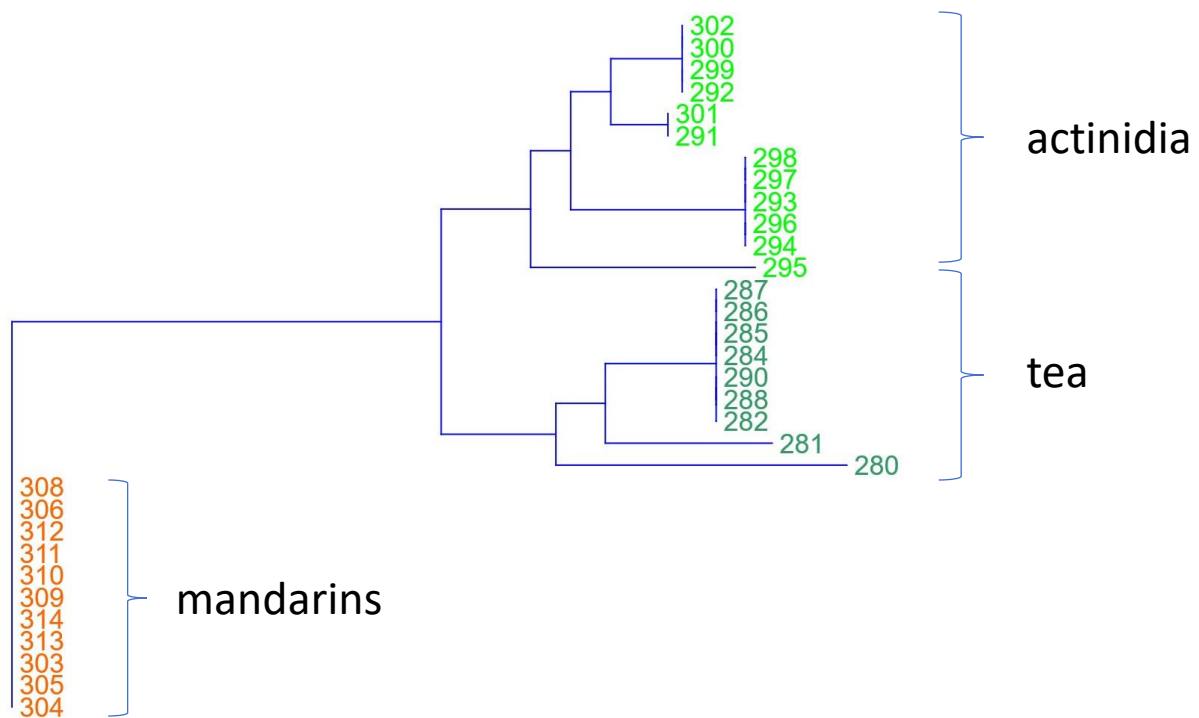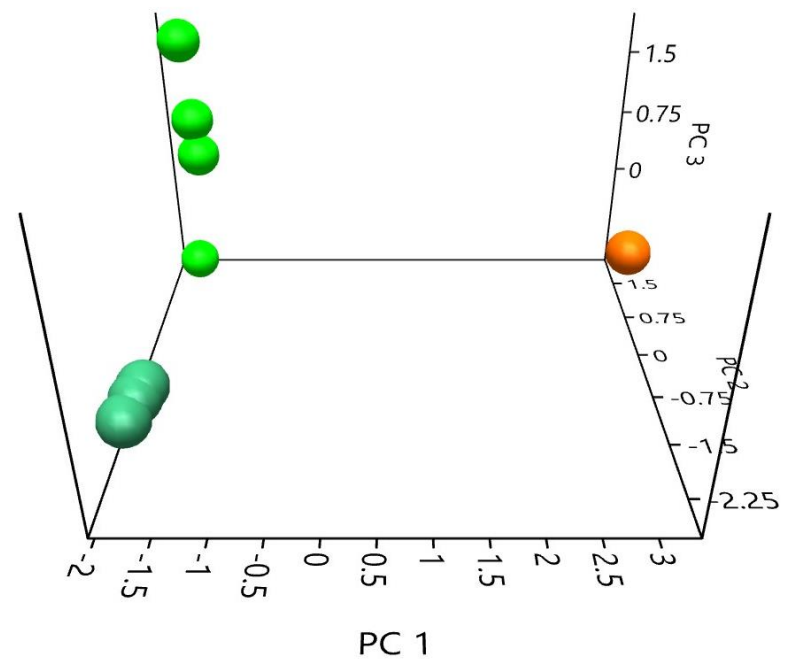

## rpl2 intron

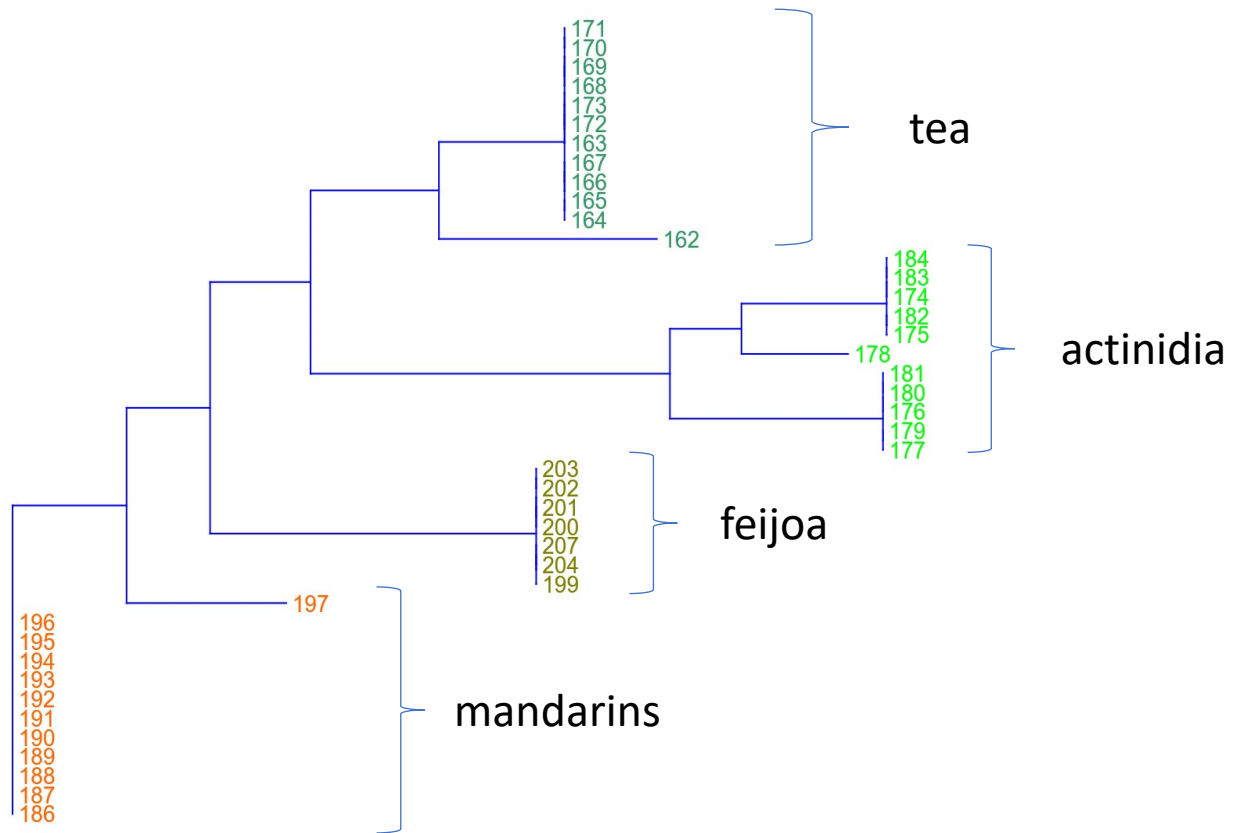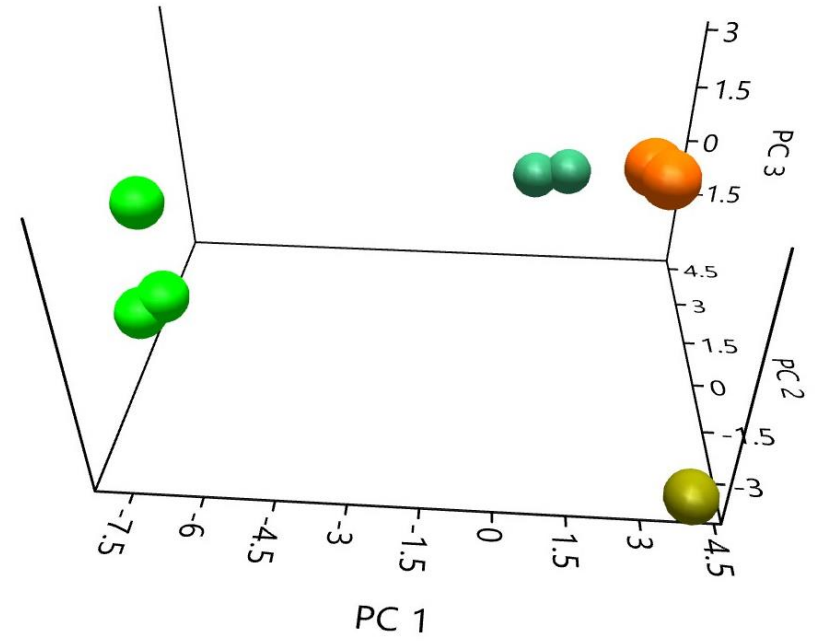

rpoC1

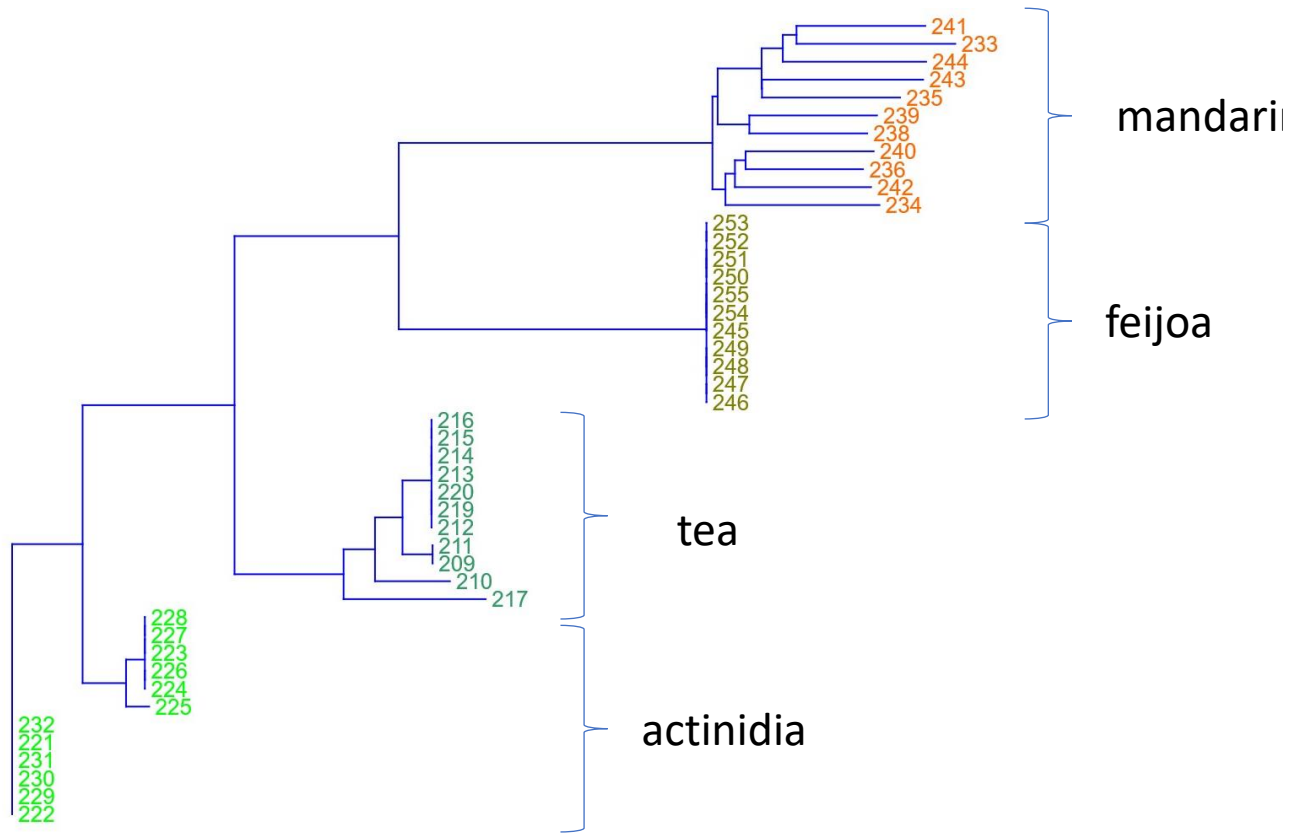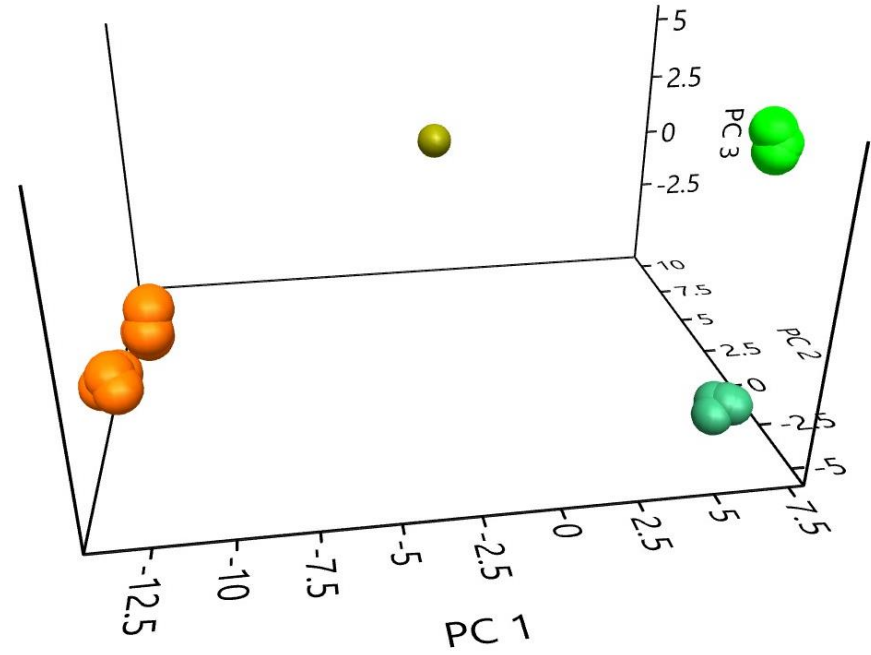

ITS-p5/ITS-u4

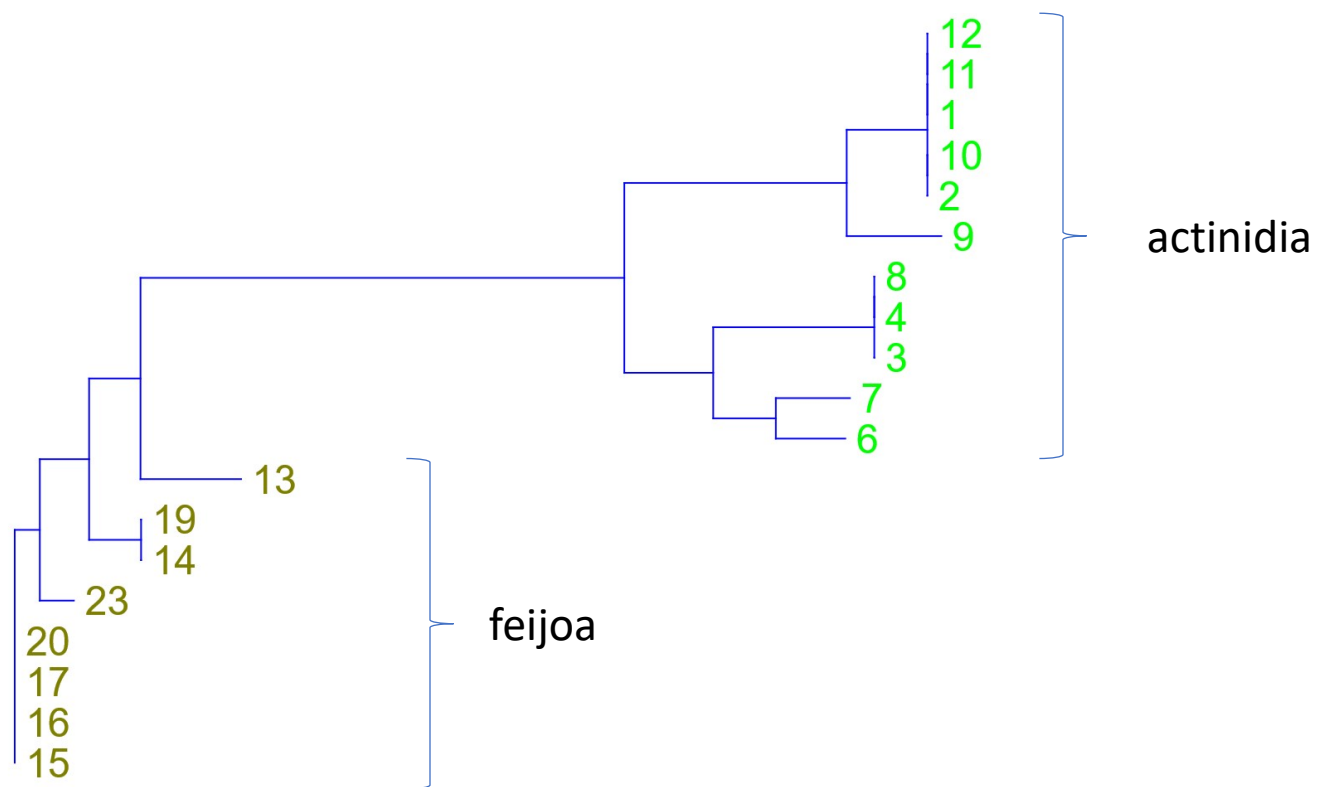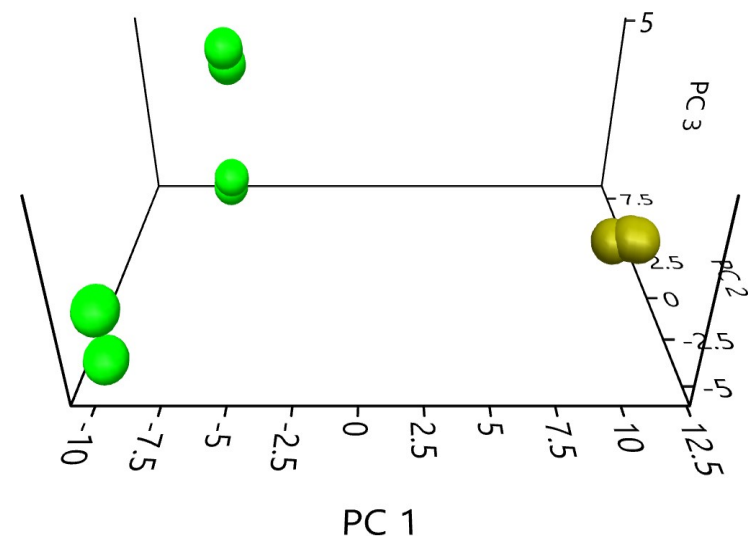

Supplement: Supplementary file 1 [file ijms-26-06921-s001.zip › ijms-3713389-supplementary.pdf]
